# Supplementary material for: Left Atrium Assessment by Speckle Tracking Echocardiography in Cryptogenic Stroke: Seeking Silent Atrial Fibrillation
Source: J Clin Med. 2021 Aug 9;10(16):3501. doi: 10.3390/jcm10163501 (PMC8397042; doi:10.3390/jcm10163501)
Supplement: Supplementary file 1 [file jcm-10-03501-s001.zip › jcm-1312243-supplementary.pdf]

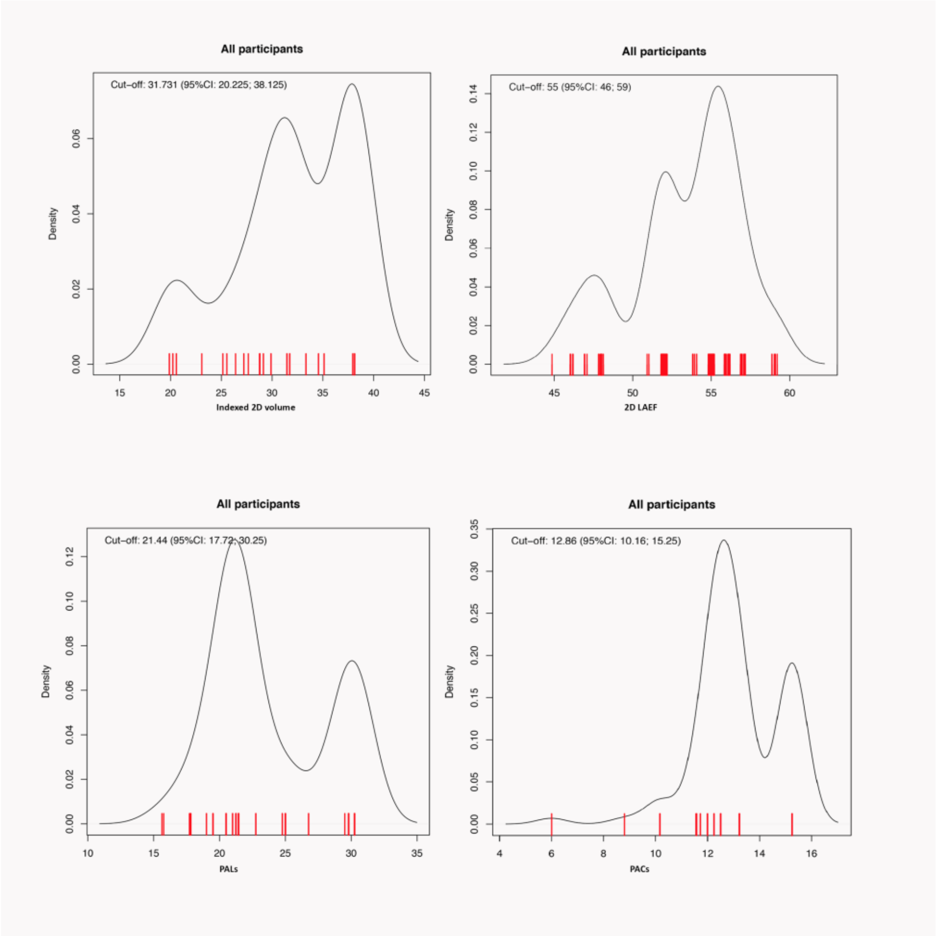

Figure S1: Bootstrapping analysis graphs for the echocardiographic parameters analysed: left atrial ejection fraction (LAEF), peak atrial longitudinal strain (PALs), and peak atrial contraction strain (PACs).
